# LINC00998 functions as a novel tumor suppressor in acute myeloid leukemia via regulating the ZFP36 ring finger protein/mammalian target of rapamycin complex 2 axis

**DOI:** 10.1080/21655979.2021.1996506

**Published:** 2021-12-02

**Authors:** Ximin Fang, Xiazhen Pan, Huirong Mai, Xiuli Yuan, Sixi Liu, Feiqiu Wen

**Affiliations:** a Department of Pediatrics, The First Affiliated Hospital of Jinan University, Guangzhou, China; b Department of Hematology-Oncology, Shenzhen Children’s Hospital, Shenzhen, China; c Department of Intensive Care Unit, The University of Hong Kong-Shenzhen Hospital, Shenzhen, China

**Keywords:** LncRNA, acute myeloid leukemia, mRNA decay, prognosis

## Abstract

Acute myeloid leukemia (AML) is a severe hematologic malignancy that threatens human health. Long non-coding RNA (lncRNA) is emerged as a key player in human cancer. Herein, we explored the role of LINC00998 in human AML. LINC00998 was significantly decreased in human AML, which was linked to relapse and poor prognosis. Stable overexpression of LINC00998 inhibited AML cell viability, colony ability, DNA synthesis rate and increased apoptosis. LINC00998 was mainly located in the cytoplasm, in which interacted with ZFP36 ring finger protein (ZFP36), a mRNA destabilizing factor, resulting in increased decay of mammalian target of rapamycin complex 2 (mTORC2), a well-known proto-oncogene in AML. Overexpression of mTORC2 partly blocked the tumor suppressive effects of LINC00998. Importantly, LINC00998 shortened *in vivo* AML cell survival in xenograft tumor model. Taken together, we found that LINC00998 is a novel tumor-inhibiting lncRNA in human AML. The dysregulation of LINC00998/ZFP36/mTORC2 axis is linked to leukemogenesis and progression.

## Introduction

Acute myeloid leukemia (AML) is the most common acute leukemia, characterized by accumulation of immature myeloid cells and hematopoietic suppression in the bone marrow [1]. At present, chemotherapy is still the main treatment, but about 70% of the patients with remission eventually relapse and develop into refractory leukemia, leading to failure of treatment and death [2]. The etiology and pathogenesis of AML are extremely complex, although the research in this field has made great progress, the 5-year survival rate is still very poor (27.4%) [3]. Hence, it is of great research value to reveal its potential pathogenesis and bringing clinical targets.

For a long time, the research on non-coding RNA (ncRNA) mainly focused on RNA less than 200 nucleotides, such as microRNA, rRNA and so on. Long non-coding RNA (lncRNA) is a type of endogenous RNA with a length of more than 200 nucleotides [4]. It has no typical start codon, promoter conserved region and open reading frame, etc., and contains a large number of stop codons, thus, it was once considered as transcriptional ‘noise’ without biological functions [5]. In recent years, the development of high-throughput experimental technology has made a great contribution to the screening and detection of functional lncRNAs related to a variety of cancer regulatory processes [6,7]. LncRNA is frequently abnormally expressed in human cancer, and is implicated in cancer initiation, development and progression [8]. For instance, lncRNA BX111887 induced by hypoxia promoted the metastasis and progression of pancreatic cancer, and its overexpression was strongly linked to poor prognosis [9]. LncRNA TINCR served as an oncogene in hepatocellular carcinoma via directly interacting with T-cell protein tyrosine phosphatase [10]. LncRNA LINC01410 was upregulated in gastric cancer, and enhanced the malignancy of gastric cancer cells via forming a feedback loop with NF-κB [11]. Although the importance of lncRNA in cancer, little is known about its role in human AML.

In the current study, we analyzed the GEPIA database (http://gepia.cancer-pku.cn/) containing tumor and normal samples from the TCGA and the GTEx databases. LINC00998 was found to be specifically and differentially expressed in AML among 32 types of human cancers. We verified its downregulation in AML in our cohort, and further explored its function and mechanism. Thus, our study aimed to explore the expression, function and clinical significance of LINC00998 in AML, and further reveal the underlying regulatory mechanism of LINC00998.

## Materials and methods

### AML samples and cell lines

80 cases of patients with *de novo* AML (32 cases with relapse) and 59 healthy controls were enrolled from January 2015 to April 2021. The diagnostic criteria for primary and recurrent AML were based on the French-American-British (FAB) and the 2016 World Health Organization classifications [12]. Lymphoprep density gradient centrifugation was applied to separate peripheral blood mononuclear cells (PBMCs) from the bone marrow samples. All patients were followed up and the written informed consent was obtained. The median survival time of AML patients in our cohort was 52.38 months. AML patient’s demographic and clinical characteristics were shown in Table 1. This study was approved by the Institutional Ethics Committee of Shenzhen Children’s Hospital. Two AML cell lines U937 and THP-1 were purchased from ATCC and cultured in RPMI-1640 medium with 10% FBS.

**Table 1.** The clinicopathological parameters of AML patients (n = 80)

Characteristics	No. (%)
Age, years	
<6	46 (57.5%)
≥6	34 (42.5%)
Gender	
Male	41 (51.3%)
Female	39 (48.7%)
White blood cells (/µL)	
<10,000	43 (53.8%)
≥10,000	37 (46.2%)
FAB classification	
M0	4 (5%)
M1	8 (10%)
M2	16 (20%)
M3	25 (31.2%)
M4	21 (26.3%)
M5	4 (5%)
M6	2 (2.5%)
Karyotype classification	
Favorable	24 (30%)
Intermediate	37 (46.3%)
Poor	14 (17.5%)
No data	5 (6.2%)

### qRT-PCR assay

The protocol was described in the previous study [13]. Total RNA was extracted by Trizol reagent (Invitrogen, USA). The PARIS Kit (Life Technologies, USA) was used for nucleocytoplasmic RNA separation as per the supplier’s instructions. Then, the highly sensitive and specific qRT-PCR was conducted using QuantiNova RT-PCR Kits with SYBR® Green I (Qiagen, Germany) for gene expression analysis. GAPDH was used as reference control. Gene relative expression was calculated by 2^−ΔΔCt^ method.

### CCK-8, EdU and soft agar assays

The protocols of functional assays were described in the previous study [13]. In short, CCK-8 assay was conducted with 10 microliter CCK-8 solution purchased from Dojindo (Japan). The absorption value at 450 nm was analyzed using an automatic microplate reader. EdU assay was used to test the DNA synthesis rate of AML cells, which was conducted by using a commercial EdU kit provided by Solarbio (China). Cell nucleus was stained by Hoechst 33342. Soft agar assay was performed using the CytoSelect 96-Well Cell Transformation Assay (Cell Biolabs Inc., USA) according the manufacturer’s protocol. Cells were cultured for one week, followed by counting and photographing,

### Detection of apoptosis

The digestive enzymes were used to prepare AML cell suspension, and then the Annexin V-FITC/PI Apoptosis Assay Kit (BD-556547, USA) was used to determine cell apoptosis. The results were recorded by flow cytometer and analyzed using Flowjo software.

### Stable LINC00998-overexpressed cell lines

The full-length of LINC00998 was cloned into pSIN-EF1α-IRES-puro lentiviral vector, followed by transfection into U937 and THP-1 cells in the presence of 6 μg/mL polybrene. 72 h later, puromycin was added into RPMI-1640 medium to screen stable clones expressing LINC00998.

### Western blot

The protocol was described in the previous study [13]. Total protein from AML cells was extracted by Radio-Immunoprecipitation Assay (RIPA) buffer, followed by addition with 1× loading dye and boiling for 10 min. Then, 20 μg protein samples were loaded into 10% dodecyl sulfate,sodium salt-polyacrylamide gel electrophoresis, transferred onto polyvinylidene fluoride membrane, and blocked with 5% defatted milk powder for 1 h at room temperature. After that, the membrane was impregnated in primary and secondary antibodies. Lastly, chemiluminescence was conducted using a highly sensitive electrochemiluminescence solution (Thermo Fisher, USA). The specific primary antibodies used in this study are as follows: anti-Cyclin D1 (ab40754, Abcam), anti-Bcl2 (ab32124, Abcam), anti-Bax (ab32503, Abcam), anti-mTORC2 (ab109081, Abcam), anti-ZFP36 (ab83579, Abcam), anti-Ki-67 (ab92742, Abcam).

### RNA pull-down and immunoprecipitation (RIP) assays

The protocol was described in the previous study [14], but with minor modifications. AML cell lysates were prepared and added with biotin-labeled LINC00998 DNA probe synthesized by Sangon (Shanghai, China), followed by incubation at room temperature for 2 h. Then, the magnetic streptavidin beads (Thermo Fisher, USA) were added into above complex and incubated at room temperature for 0.5 h. The enriched protein was eluted for western blot assay. RIP assay was carried out by using Magna RIP™ RNA-Binding Protein Immunoprecipitation Kit (Promega, USA) according to the manufacturer’s instructions.

### Animal study

10 nude mice were randomly divided into 2 groups (n = 5 per group). LINC00998-overexpressing U937 cells were subcutaneously injected into each mice and grown for 4 weeks. The volume of the subcutaneous tumor was weekly measured.

Then, all mice were sacrificed and tumor tissues were collected for qRT-PCR and western blot assays. Cell apoptosis was conducted using TUNEL Apoptosis Detection Kit based on supplier’s instructions (YEASEN, China).

### Statistical analysis

Student’s t test was used to analyze the difference between two groups. All functional experiments were repeated independently at least 3 times. Figures were plotted by Graphpad 7.0 software and statistics were done using SPSS 18.0 software. *P* < 0.05 was considered to have statistical difference.

## Results

Herein, we explored the role of LINC00998 in AML. We found that LINC00998 was significantly decreased in human AML. Next, we performed a series of functional and animal assays, found that LINC00998 inhibited AML progression through regulation of mTORC2 mRNA stability via binding to ZFP36. Our data uncover the importance of LINC00998 in AML, and provide new insights into the pathogenesis of AML.

### LINC00998 is significantly downregulated in human AML

**Figure 1.**
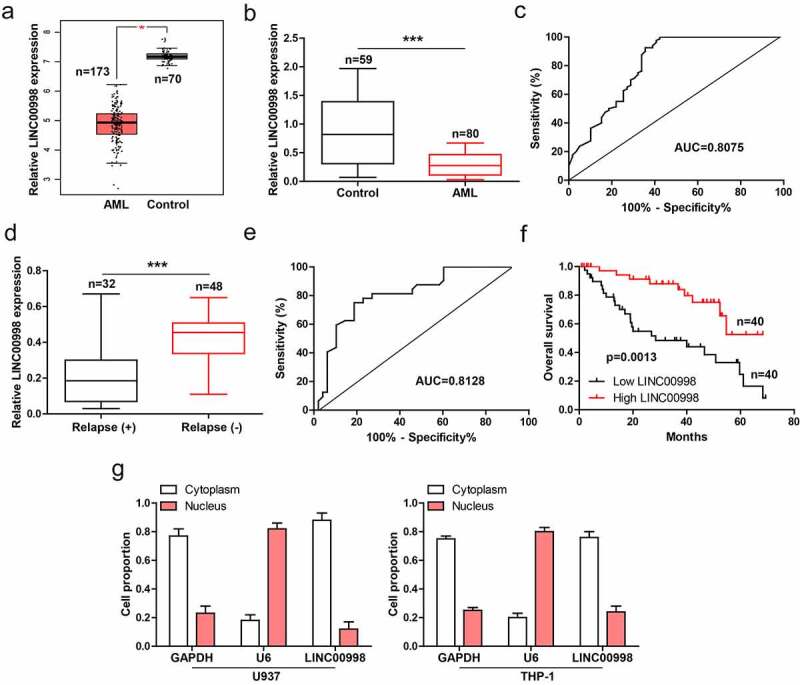
LINC00998 is lowly expressed in human AML. (a) GEPIA database showing the expression of LINC00998 in AML. (b, c) qRT-PCR analysis of LINC00998 in PBMCs from 80 AML patients and 59 healthy controls, followed by ROC curve analysis. (d, e) qRT-PCR analysis of LINC00998 in PBMCs from AML patients with or without relapse, followed by ROC curve analysis. (f) The survival curve of AML patients with low or high LINC00998 level based on median LINC00998 expression (Hazard Ratio = 3.161, 95% confidence interval = 1.565 ~ 6.386). (g) qRT-PCR analysis of LINC00998 level in nuclear and cytoplasmic fragments of U937 and THP-1 cells. ***P < 0.001

The GEPIA data showed that LINC00998 was notably decreased in AML (n = 173) as compared to normal control (n = 70) (Figure 1(a)). We then collected PBMCs from 80 AML patients and 59 healthy controls, and conducted qRT-PCR analysis. The results showed that LINC00998 expression in AML was significantly less than than in healthy control (Figure 1(b)), with an area under ROC curve (AUC) of 0.8075 (95%CI: 0.7300 to 0.8850) (Figure 1(c)), hinting the level of LINC00998 can effectively predict AML. Moreover, patients with relapse had lower LINC00998 than those without relapse (Figure 1(d)), with an AUC of 0.8128 (95%CI: 0.7172 to 0.9085) (Figure 1(e)). Importantly, low LINC00998 was positively correlated with shorter survival time of AML patients (Figure 1(f)). Next, by analyzing lncATLAS (https://lncatlas.crg.eu/) database, we found that LINC00998 was a cytoplasmic lncRNA (Figure S1), the qRT-PCR results verified the cytoplasmic location of LINC00998 in AML cells (Figure 1(g)).

### LINC00998 inhibits AML cell survival and induces apoptosis

**Figure 2.**
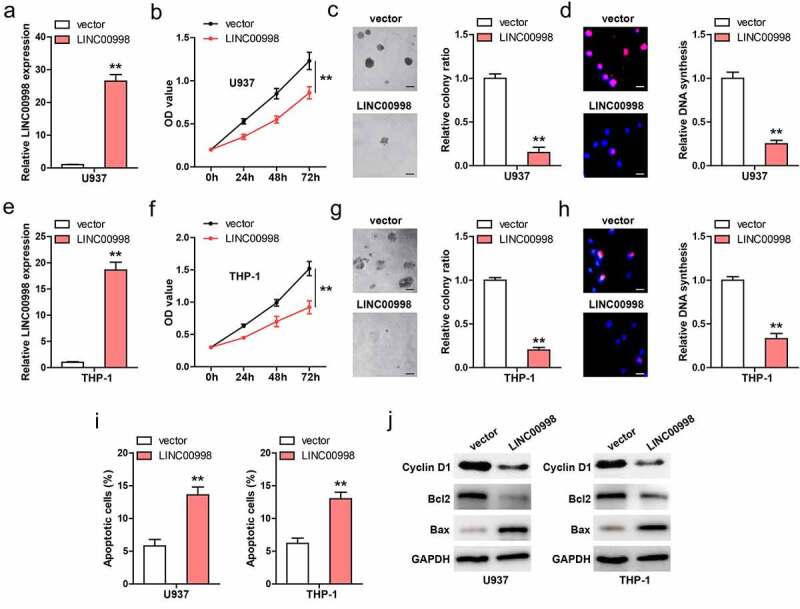
LINC00998 inhibits AML cell malignant behaviors. (a) qRT-PCR verifying the overexpression of LINC00998 in U937 cells. (b–d) CCK-8, colony formation and EdU assays testing cell viability, colony and DNA synthesis rate of U937 cells after LINC00998 overexpression. (e–h) The effect of LINC00998 overexpression on cell viability, colony and DNA synthesis rate. (i) Flow cytometry analyzing cell apoptosis after LINC00998 overexpression. (j) The indicated protein levels in LINC00998-overexpressing AML cell lines. Scale bar = 50 μm, **P < 0.01

We established stable LINC00998-overexpressed U937 cell lines (Figure 2(a)), and conducted a series of functional assays. CCK-8 results showed that cell viability was significantly weakened after LINC00998 overexpression (Figure 2(b)). Moreover, less colony was formed in LINC00998-overexpressed cells as compared to control cells (Figure 2(c)). And DNA synthesis rate was slowed down by LINC00998 overexpression (Figure 2(d)). To further validate the effect of LINC00998 in AML, we generated stable LINC00998-overexpressed THP-1 cell lines (Figure 2(e)), and found that LINC00998 also inhibited THP-1 cell viability, colony formation and DNA synthesis rate (Figure 2(f–h)). In addition, the flow cytometry results showed that the number of apoptotic cells increased after LINC00998 overexpression in both U937 and THP-1 cells (Figure 2(i), Figure S2). In line with these phenotype, Cyclin D1 (proliferative factor) and Blc2 (anti-apoptotic factor) were decreased, while Bax (apoptotic factor) was increased in LINC00998-overexpressed AML cells (Figure 2(j)).

### mTORC2 is the downstream target of LINC00998

**Figure 3.**
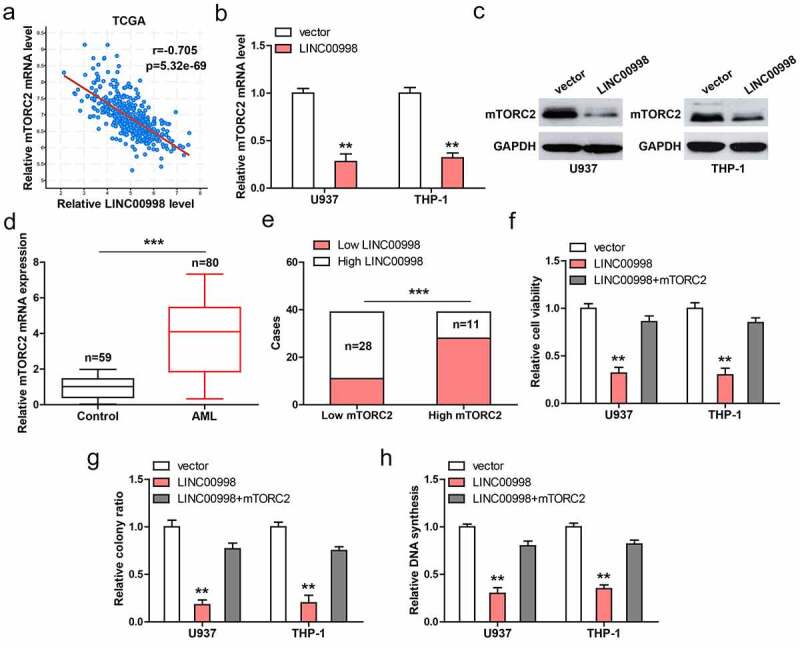
LINC00998 inhibits mTORC2 level. (a) The correlation between LINC00998 and mTORC2 mRNA in TCGA AML database. (b, c). The mRNA and protein levels of mTORC2 after LINC00998 overexpression. (d) qRT-PCR analysis of mTORC2 mRNA levels in PBMCs from 80 AML patients and 59 healthy controls. (e) The correlation between LINC00998 and mTORC2 mRNA in AML. F-H. Cell viability, colony and DNA synthesis in LINC00998-overexpressed U937 and THP-1 cells with mTORC2 overexpression. **P < 0.01, ***P < 0.001

Through analyzing TCGA database, we found that LINC00998 was strongly negatively correlated with mTORC2 in AML (r = −0.705) (Figure 3(a)). Consistently, mTORC2 mRNA and protein levels were dramatically reduced in LINC00998-overexpressed U937 and THP-1 cells (Figure 3(b,c)). High mTORC2 was observed in PBMCs from AML patients compared to those from healthy controls (Figure 3(d)). 70% of AML patients with high LINC00998 had low mTORC2 level (Figure 3(e)). Functionally, the attenuated cell malignant behaviors caused by LINC00998 overexpression were significantly rescued after mTORC2 overexpression (Figure 3(f–h)).

### LINC00998 directly binds to ZFP36 and reduces mTORC2 mRNA stability

**Figure 4.**
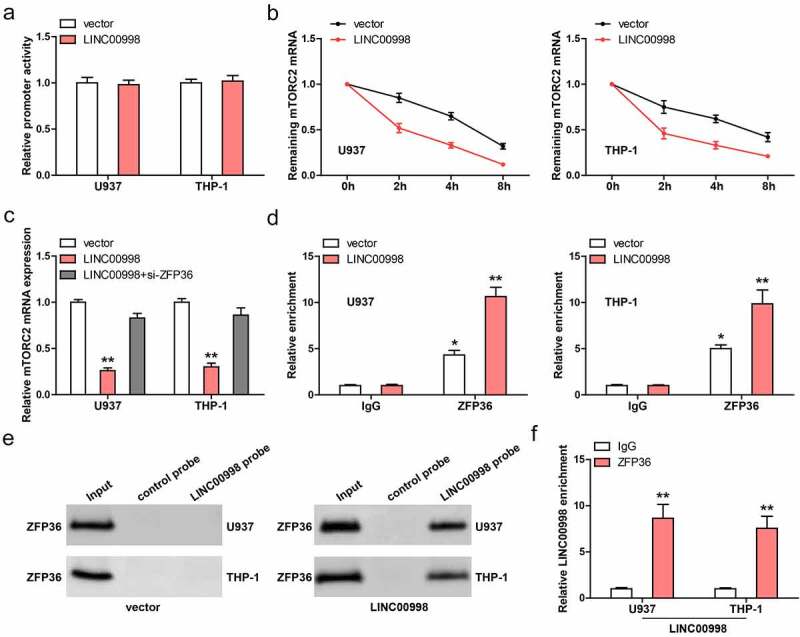
LINC00998 enhances ZFP36-mediated mTORC2 mRNA decay. (a) The promoter activity of mTORC2 affected by LINC00998. (b) qRT-PCR analysis of mTORC2 mRNA level at the indicated time in LINC00998-overexpressed AML cells treated with Actinomycin D. (c) qRT-PCR testing the effect of ZFP36 silencing on mTORC2 expression in LINC00998-overexpressed AML cells. (d) The enrichment of mTORC2 3`-UTR by ZFP36 antibody. (e) RNA pull-down assay using LINC00998 probe, followed by western blot analysis of ZFP36 level. (f) The enrichment of LINC00998 by ZFP36 antibody. *P < 0.05, **P < 0.01

We then tested how LINC00998 affected mTORC2 mRNA level. As shown in Figure 4(a), the promoter activity of mTORC2 was not altered after LINC00998 overexpression in these two AML cells. However, the half-life of mTORC2 mRNA was significantly reduced after LINC00998 overexpression when the cells were treated with Actinomycin D, a transcription suppressor (Figure 4(b)). Importantly, the decreased mTORC2 mRNA caused by LINC00998 was notably blocked by silencing of ZFP36 (Figure 4(c)), a mRNA destabilizing factor, revealing that ZFP36 is responsible for the downregulation of mTORC2 mediated by LINC00998. Further, RIP assay showed that mTORC2 3`-UTR was abundantly enriched by ZFP36 antibody (Figure 4(d)), and this effect was enhanced in LINC00998-overexpressed AML cells (Figure 4(d)). Next, we synthesized biotin-labeled LINC00998 probe and conducted RNA pull-down assay. As shown in Figure 4(e), the endogenous ZFP36 protein was undetectable in cell lysates pulled down by LINC00998 probe, but was detectable after LINC00998 overexpression, which may be the low content of endogenous LINC00998 in AML cells. Reciprocally, LINC00998 was significantly enriched by ZFP36 antibody in LINC00998-overexpressed cells, as illustrated by RIP assay (Figure 4(f)).

### *LINC00998 inhibits AML cell survival and induces apoptosis* in vivo

**Figure 5.**
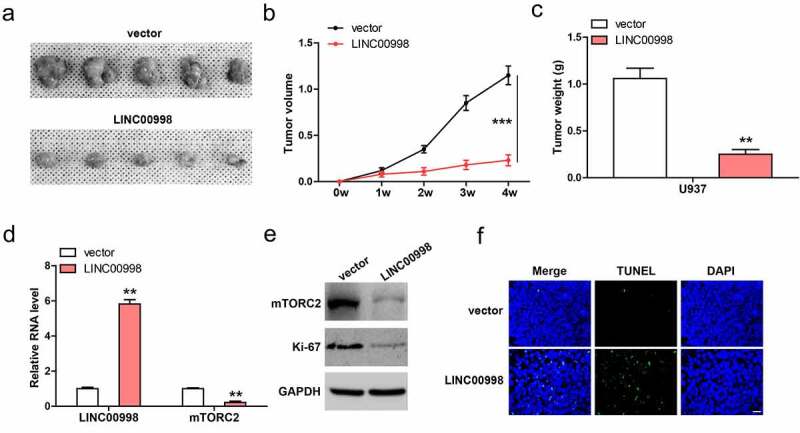
LINC00998 inhibits tumor growth *in vivo*. (a–c). Tumor volume, weight and image in control and LINC00998-overexpressing groups. (d) qRT-PCR analysis of LINC00998 and mTORC2 levels in the indicated two groups. (e) Western blot analyzing mTORC2 and Ki-67 levels in the indicated two groups. (f) TUNEL staining testing cell apoptosis in the indicated two groups. Staining of nucleus with DAPI. Scale bar = 50 μm, **P < 0.01, ***P < 0.001

Lastly, we conducted subcutaneous injection of U937 cells into nude mice. As shown in Figure 5(a–c), tumor volume and weight in LINC00998-overexpressed group were significantly less than those in control group. Moreover, mTROC2 mRNA and protein levels were dramatically decrease in LINC00998-overexpressed tumor tissues (Figure 5(d)), accompanied by the decrease of Ki-67 proliferation index (Figure 5(e)). TUNEL staining showed that LINC00998-overexpressed tissues had more apoptotic cells than control tissues (Figure 5(f)).

## Discussion

The etiology of AML is still not fully understood and ongoing research is underway. In this study, we described a novel tumor-inhibiting factor in AML. Endogenous LINC00998 was drastically reduced in human AML, linking to aggressive clinical features and dismal prognosis. The functional assays and nude mice study showed that LINC00998 inhibited AML cell survival *in vitro* and *in vivo*. Further, we revealed the mechanism of LINC00998 inhibiting AML progression. In detail, LINC00998 served as a partner of ZFP36, facilitating the binding of ZFP36 on mTORC2 mRNA 3`-UTR, leading to the decay of mTORC2 mRNA. Thus, our data suggest that LINC00998 is a novel negative regulator of AML tumorigenesis, and also highlight the functional implication of lncRNA as a protein binding partner.

Emerging evidence demonstrates that lncRNA is expected to be novel biomarker for early diagnosis, prognostic value and therapeutic target, due to the relative tissue-specific expression [15,16]. For example, by conducting transcriptome sequencing, Silva-Fisher et al found that lncRNA RAMS11 was significantly overexpressed in metastatic colorectal cancer, and it increased the resistance to topoisomerase inhibitors, which could be used as a biomarker and therapeutic target for metastatic colorectal cancer patients [17]. Xie et al found a squamous cell carcinoma (SCC)-specific lncRNA, LINC01503, which was highly expressed in SCC and associated with shorter survival time. LINC01503 promoted SCC cell aggressive phenotype and targeting of LINC01503 reduced the growth of xenograft tumors [18]. Recently, Fan et al. characterize a lncRNA CISAL forming a DNA-RNA triplex and regulating BRCA1 transcription, which predicted sensitivity to cisplatin and could be utilized as a treatment efficacy indicator [19]. Here, we analyzed the GEPIA database and found LINC00998 to be specifically and differentially expressed in AML among 32 types of human cancers. AML patients with high LINC00998 had longer survival time than patients with low LINC00998, and LINC00998 was significantly decreased in human AML (AUC = 0.8075) and recurrent tissues (AUC = 0.8128), suggesting that LINC00998 is tissue-specifically expressed in AML and can be used as a promising biomarker for AML diagnosis, prognosis and relapse. However, there are many factors that need to be controlled before LINC00998 becomes a useful marker, for example, the tissue samples in our study were relatively insufficient and subsequent studies should make up for this, so as to better clarify the clinical value of LINC00998.

LncRNA plays a role in a variety of mechanisms, among which its role as a protein binding partner has attracted increasing attention [20]. The most well-known lncRNA is HOTAIR, a oncogene in human cancer [21], its 5ʹ domain could bind polycomb repressive complex 2, while its 3ʹ domain bound the LSD1/CoREST/REST complex, thereby controlling target gene expression via specifying the pattern of histone modifications [22]. Besides, SNHG11 was shown to be upregulated in colorectal cancer and blocked the interaction between pVHL and HIF-1α via binding to HIF-1α, preventing HIF-1α ubiquitination and degradation [23]. In this study, we found that LINC00998 was able to directly bind to ZFP36 and increased the degradation of mTORC2 mRNA. ZFP36 is a RNA-binding protein that reduced mRNA stability via binding to AU-rich elements on the 3ʹ UTR of target gene. Consistent with a previous report identifying mTORC2 as a target of ZFP36 [24], our RIP data showed that mTORC2 3ʹ UTR was abundantly enriched by ZFP36 antibody, which was significantly increased after LINC00998 overexpression, suggesting that LINC00998 may be a key auxiliary for ZFP36 in AML cells. mTORC2 is a well-documented oncogene in cancer [25], including AML [26]. We verified the upregulation of mTORC2 in human AML and overexpression of mTORC2 blocked the reduced malignant behaviors of AML cells induced by LINC00998 overexpression, revealing that LINC00998 functions as a tumor suppressor via targeting mTORC2. Consistent with our results, a recent study showed that LINC00998 was decreased in human malignant glioma and negatively regulated by miR-34c-5p [27]. Whether LINC00998 is a pan-tumor suppressor gene needs further study and discussion.

In addition, some lncRNAs have been proven to be a key regulator in AML progression, such as ANRIL [28], HOTTIP [29], SATB1-AS1 [30], etc. Further studies are needed to clarify whether these lncRNAs interact with each other to form a huge regulatory network that accurately controls the occurrence and development of AML, and whether several combinations of these lncRNAs can better diagnose and predict AML.

## Conclusion

Our work unveil the critical role of LINC00998 inhibiting AML growth via the ZFP36/mTORC2 axis. Restoration of LINC0998 may be a potential therapeutic protocol for AML patients.

## Supplementary Material

Supplemental Material

## Highlights

1. LINC00998 is significantly downregulated in human AML, which links to poor prognosis.

2. Overexpression of LINC00998 inhibits AML cell survival and induces apoptosis.

3. LINC00998 reduces mTORC2 mRNA stability.

4. LINC00998 directly binds to ZFP36 and promotes mTORC2 mRNA decay.

5. Restoration of LINC00998 *in vivo* represses tumor growth.
