# Supplementary material for: LINC00998 functions as a novel tumor suppressor in acute myeloid leukemia via regulating the ZFP36 ring finger protein/mammalian target of rapamycin complex 2 axis
Source: Bioengineered. 2021 Dec 2;12(2):10363–72. doi: 10.1080/21655979.2021.1996506 (PMC8810020; doi:10.1080/21655979.2021.1996506)
Supplement: Supplemental Material [file KBIE_A_1996506_SM5402.doc]

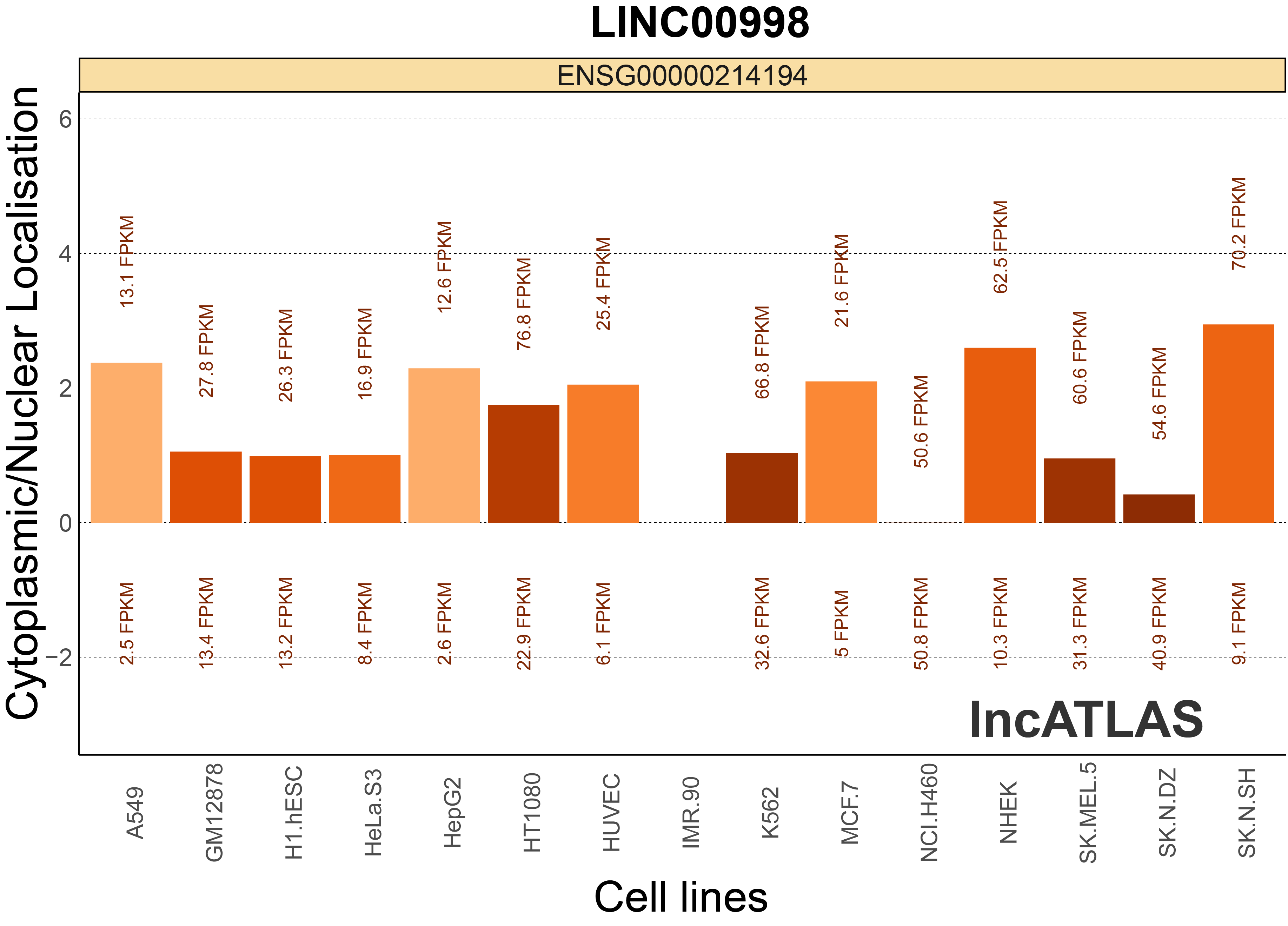


**Figure S1**. LncATLAS predicting the location of LINC00998 in different cell lines.


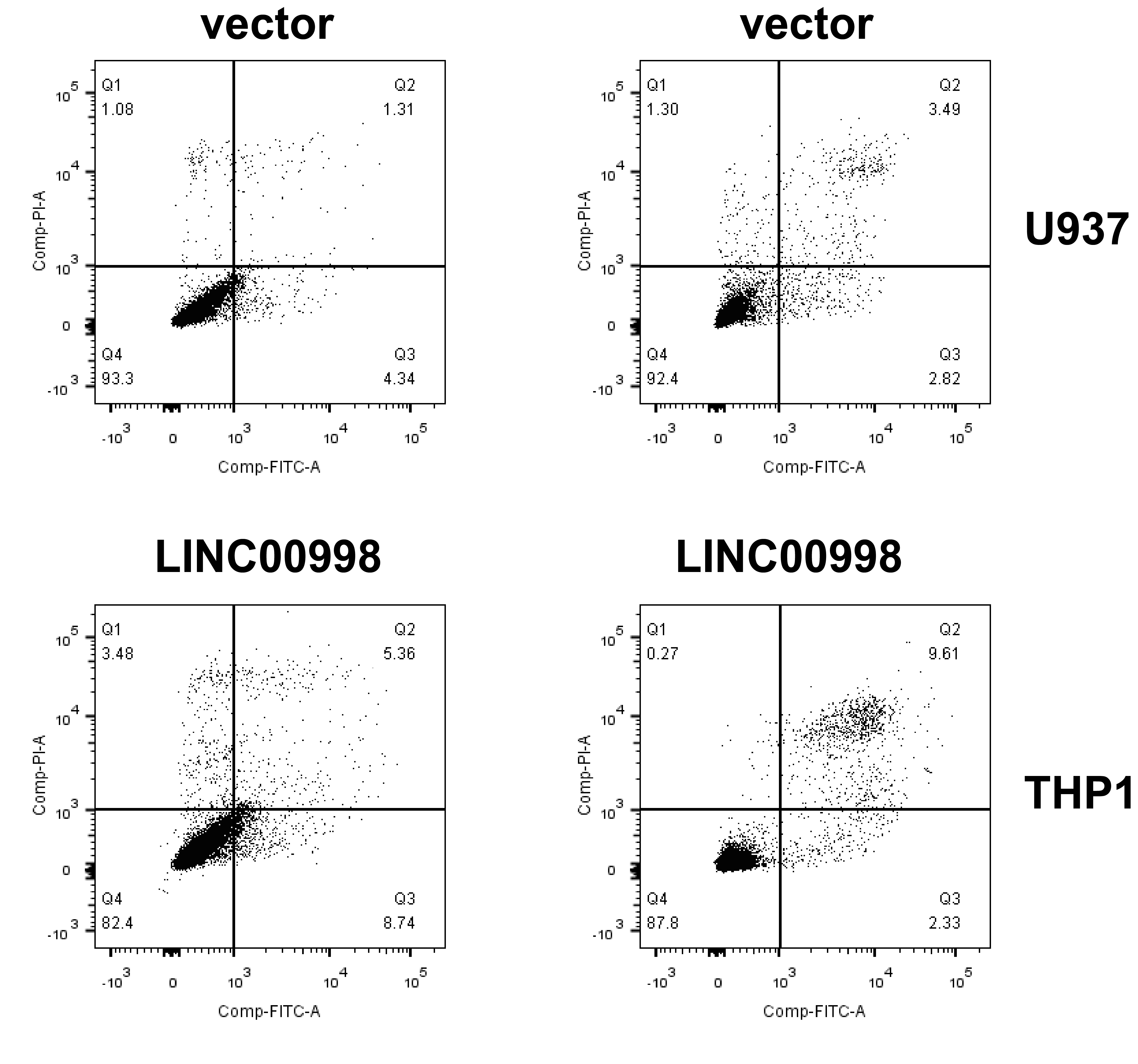


**Figure S2.** Flow cytometry analyzing cell apoptosis after LINC00998 overexpression.
